# Rapidly Progressive Femoral Head Destruction Following a Single Intra-Articular Corticosteroid Injection

**DOI:** 10.7759/cureus.117608

**Published:** 2026-10-07

**Authors:** Alexandria Zarilla, Aviva Grimmett, Victoria M Estevez, John Berra

**Affiliations:** 1 School of Medicine, Lake Erie College of Osteopathic Medicine, Bradenton, USA; 2 Orthopedic Surgery, Orthopedic Center of Florida, Fort Myers, USA

**Keywords:** avascular necrosis, femoral head collapse, femoral head destruction, intra-articular corticosteroid injection, osteonecrosis, rapidly progressive osteoarthritis, total hip arthroplasty

## Abstract

Osteonecrosis of the femoral head is a well-recognized complication of corticosteroid exposure but it is more commonly associated with prolonged systemic therapy or high cumulative doses. Rapid femoral head destruction following a single intra-articular corticosteroid injection is uncommon. We present the case of a 47-year-old woman with severe right hip osteoarthritis and a remote history of right hip arthroscopy who underwent a fluoroscopy-guided intra-articular injection containing 80 milligrams (mg) of triamcinolone after persistent symptoms despite conservative management. Initial radiographs demonstrated severe osteoarthritis with superior femoral head flattening but without radiographic evidence of frank femoral head collapse or subchondral lucency. Although the patient experienced transient relief, repeat imaging 10 days after injection demonstrated interval femoral head deformity. Her symptoms and functional limitations progressed rapidly, and by approximately six weeks post-injection, imaging demonstrated near-complete destruction of the femoral head. Given the severity of her pain, functional decline, and rapid structural deterioration, she underwent total hip arthroplasty with subsequent improvement in pain and ambulatory function. The temporal relationship between corticosteroid injection and rapid femoral head destruction is notable; however, the absence of pre-injection magnetic resonance imaging (MRI) and histopathologic confirmation prevents determination of the underlying mechanism or establishment of a causal relationship. Pre-existing occult osteonecrosis, subchondral insufficiency fracture, and rapidly progressive osteoarthritis remain possible contributing or alternative processes. This case highlights the importance of recognizing unexpected clinical deterioration following intra-articular hip corticosteroid injections and considering advanced imaging when symptoms progress rapidly or are disproportionate to radiographic findings.

## Introduction

Osteonecrosis is characterized by ischemic death of bone tissue resulting from disruption of its vascular supply. The femoral head is one of the most commonly affected sites, and progressive osteonecrosis can result in subchondral fracture, articular surface collapse, secondary osteoarthritis, and eventual joint destruction [1]. Early-stage osteonecrosis may not be apparent on plain radiographs because the structural changes detectable by radiography, such as sclerosis, cystic changes, subchondral fracture, and femoral head collapse, develop late in the disease course [2]. Therefore, magnetic resonance imaging (MRI) is considered the gold standard for detecting early osteonecrosis because of its high sensitivity and specificity and should be considered when clinical suspicion persists despite nondiagnostic radiographs [1]. 

Osteonecrosis of the femoral head may result from traumatic or nontraumatic causes, with corticosteroid exposure representing one of the most commonly recognized nontraumatic risk factors [3]. The femoral head is particularly susceptible to ischemic injury because its blood supply is predominantly derived from branches of the medial femoral circumflex artery with relatively limited collateral circulation [4]. Although the pathogenesis of corticosteroid-associated osteonecrosis remains incompletely understood, proposed mechanisms include abnormalities in lipid metabolism and adipocyte hypertrophy, increased intraosseous pressure, endothelial dysfunction and thrombosis, impaired microvascular perfusion, and abnormalities in osteoblast and osteocyte function [5]. Collectively, these mechanisms may compromise femoral head perfusion and contribute to osteonecrotic changes. 

Corticosteroid-associated osteonecrosis has traditionally been associated with systemic therapy, with the risk increasing with higher doses and longer duration of exposure [5]. In contrast, rapidly progressive osteonecrosis and femoral head destruction following a single intra-articular corticosteroid injection have rarely been reported [6,7]. Rapidly progressive osteoarthritis of the hip is a distinct destructive process characterized by rapid joint-space loss, with some cases progressing to femoral head and acetabular destruction [8]. However, the relationship between intra-articular corticosteroid injections and rapidly progressive hip destruction remains uncertain [9]. This case describes rapid progression of femoral head destruction following a single intra-articular corticosteroid injection in a patient with pre-existing osteoarthritis in whom osteonecrosis was not definitively established. It highlights the diagnostic uncertainty surrounding rapidly destructive hip disease and the importance of considering advanced imaging when symptoms progress rapidly or are disproportionate to radiographic findings.

## Case presentation

A 47-year-old woman with a medical history of hypertension and anxiety disorder as well as an orthopedic history of right hip arthroscopy with labral repair and iliotibial band release 11 years prior presented to pain management with a two-month history of progressive right groin pain. She reported no history of prior hip fracture or dislocation and denied significant alcohol use. The pain was described as sharp, throbbing, and grinding, aggravated by walking, standing, and working. The pain radiated anteriorly into the thigh to the level of the right knee. The physical exam demonstrated a positive log-roll test, with pain reproduced during passive internal and external rotation of the hip and mildly decreased range of motion of the right hip in the supine position. 

Initial radiographs demonstrated lateral subluxation with superior migration of the femoral head and severe osteoarthritis of the right hip, with superior femoral head flattening but without radiographic evidence of frank collapse or subchondral lucency (Figure 1). Pre-injection MRI was not obtained; therefore, the etiology of the pre-existing femoral head flattening could not be definitively characterized. Her symptoms persisted despite conservative management including activity modification, nonsteroidal anti-inflammatory medications, muscle relaxers, chiropractic care, and massage therapy. She subsequently elected to undergo a fluoroscopy-guided intra-articular hip injection consisting of 80 mg of triamcinolone, 1.5 milliliters (mL) of 0.5% ropivacaine, and 1.5 mL of 2% lidocaine.

**Figure 1:**
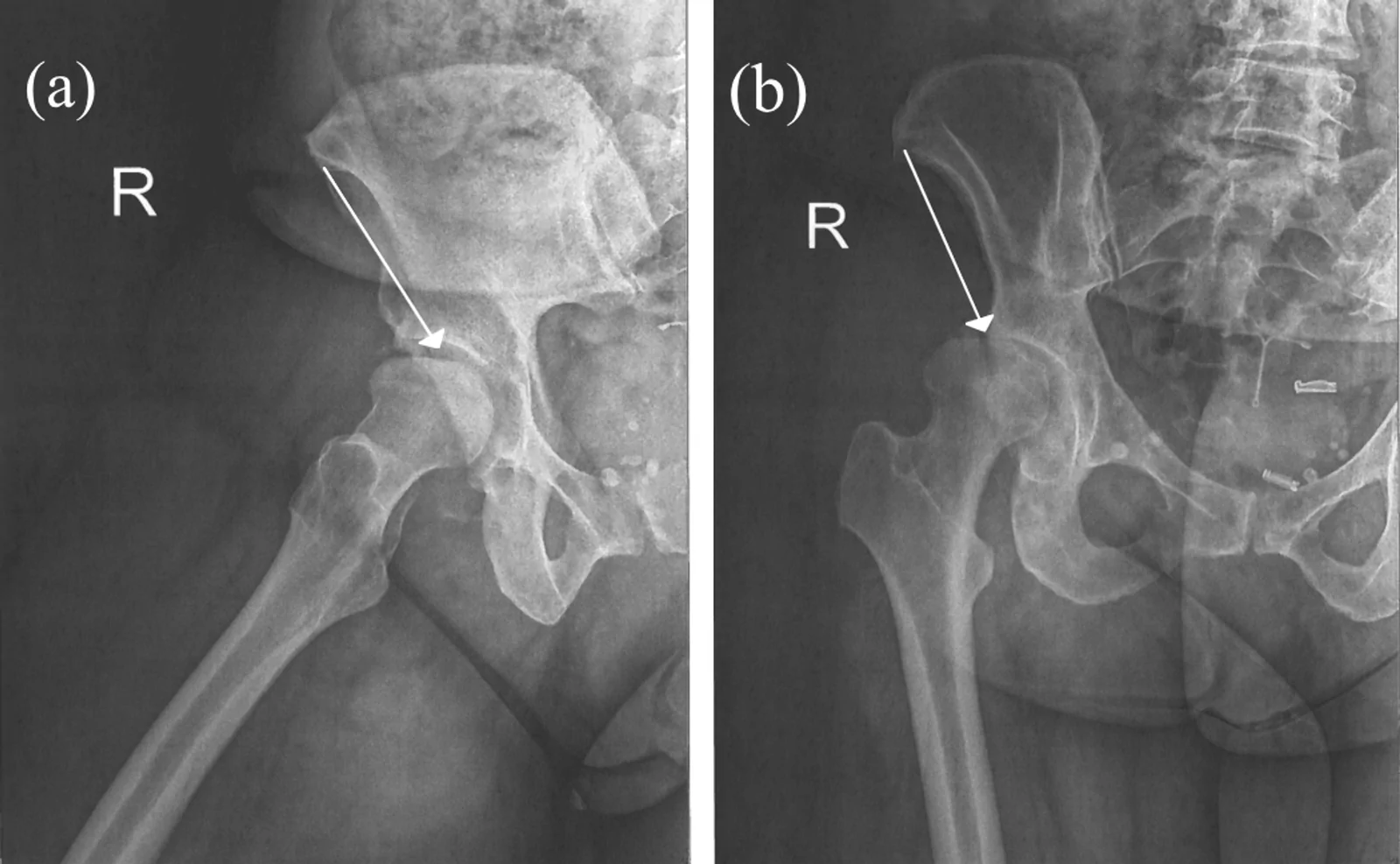
Initial radiographs of the right hip prior to intra-articular corticosteroid injection (a) Frog-leg lateral radiograph of the right hip and (b) anteroposterior radiograph of the right hip demonstrating severe osteoarthritis with joint-space narrowing and flattening of the superior femoral head (arrows), without radiographic evidence of frank femoral-head collapse or subchondral lucency.

The patient initially experienced transient pain relief following the injection. At her orthopedic follow-up 10 days after the injection, she reported worsening right groin pain and associated weakness. Repeat examination demonstrated positive flexion, abduction, and external rotation (FABER) findings; a positive C-sign, in which the patient cupped her hand around the hip to localize deep hip pain; and positive flexion, adduction, and internal rotation (FADIR) findings. Radiographs obtained at this 10-day follow-up demonstrated interval deformity of the right femoral head compared with the pre-injection imaging (Figure 2).

**Figure 2:**
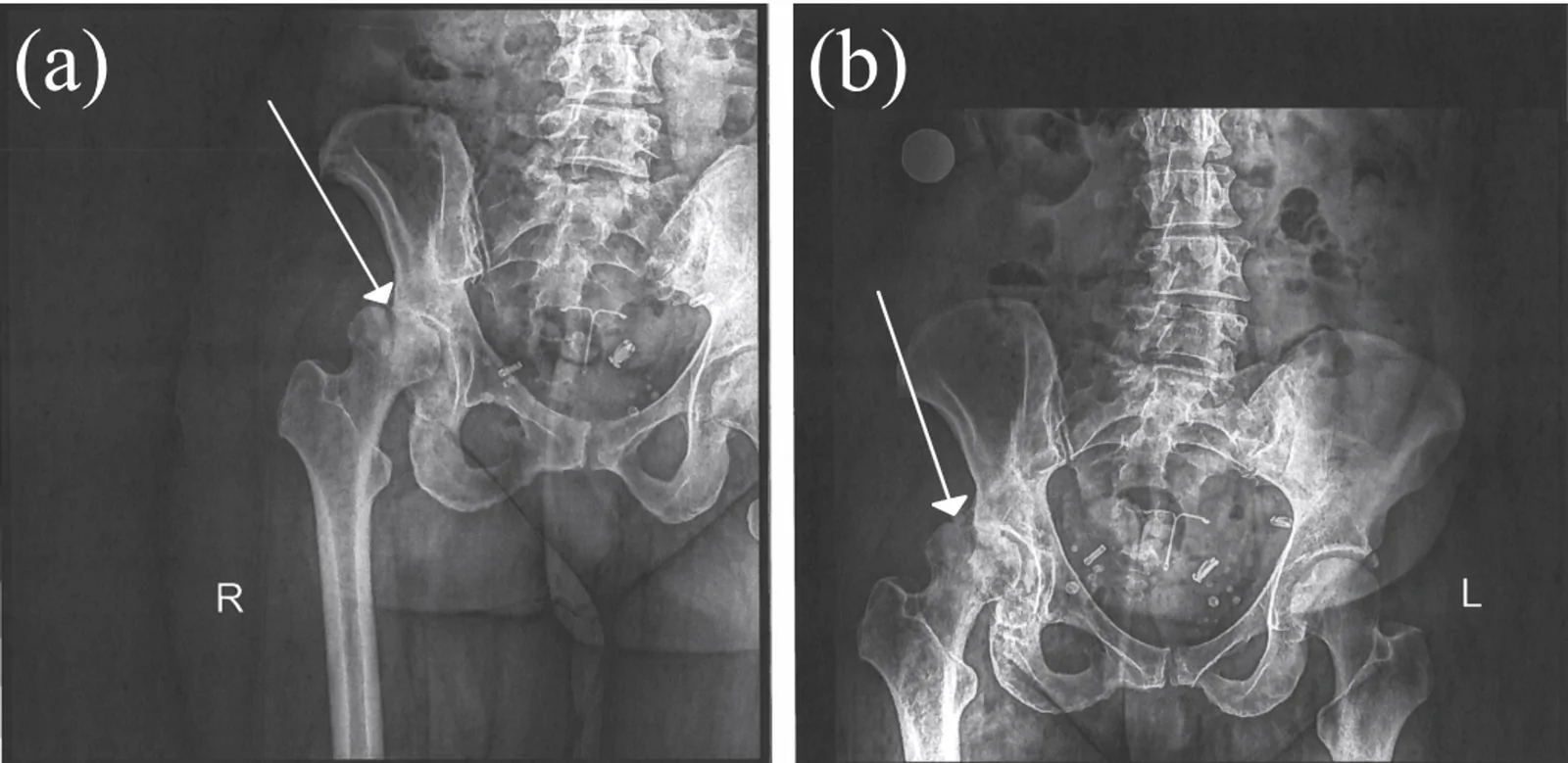
Right hip radiographs 10 days following intra-articular corticosteroid injection (a) Anteroposterior radiograph of the right hip and (b) anteroposterior radiograph of the pelvis demonstrating interval deformity and early structural deterioration of the right femoral head (arrows) compared with the pre-injection radiographs.

Given the interval radiographic changes and worsening symptoms, close follow-up was arranged. At approximately six weeks after the injection, she returned with substantially worsening pain and difficulty with ambulation. Repeat radiographs demonstrated rapidly progressive destruction and near-complete degradation of the right femoral head (Figure 3). Computed tomography (CT) confirmed the extensive femoral head destruction and additionally demonstrated a moderate-sized right hip joint effusion with prominent diffuse capsular calcifications and superior extension into the iliopsoas bursa. Due to the rapid progression of her symptoms and structural deterioration, she was provided crutches and instructed to remain non-weight bearing while awaiting surgical intervention. Given the recent intra-articular corticosteroid injection, the patient was initially advised that total hip arthroplasty would ideally be delayed for approximately three months.

**Figure 3:**
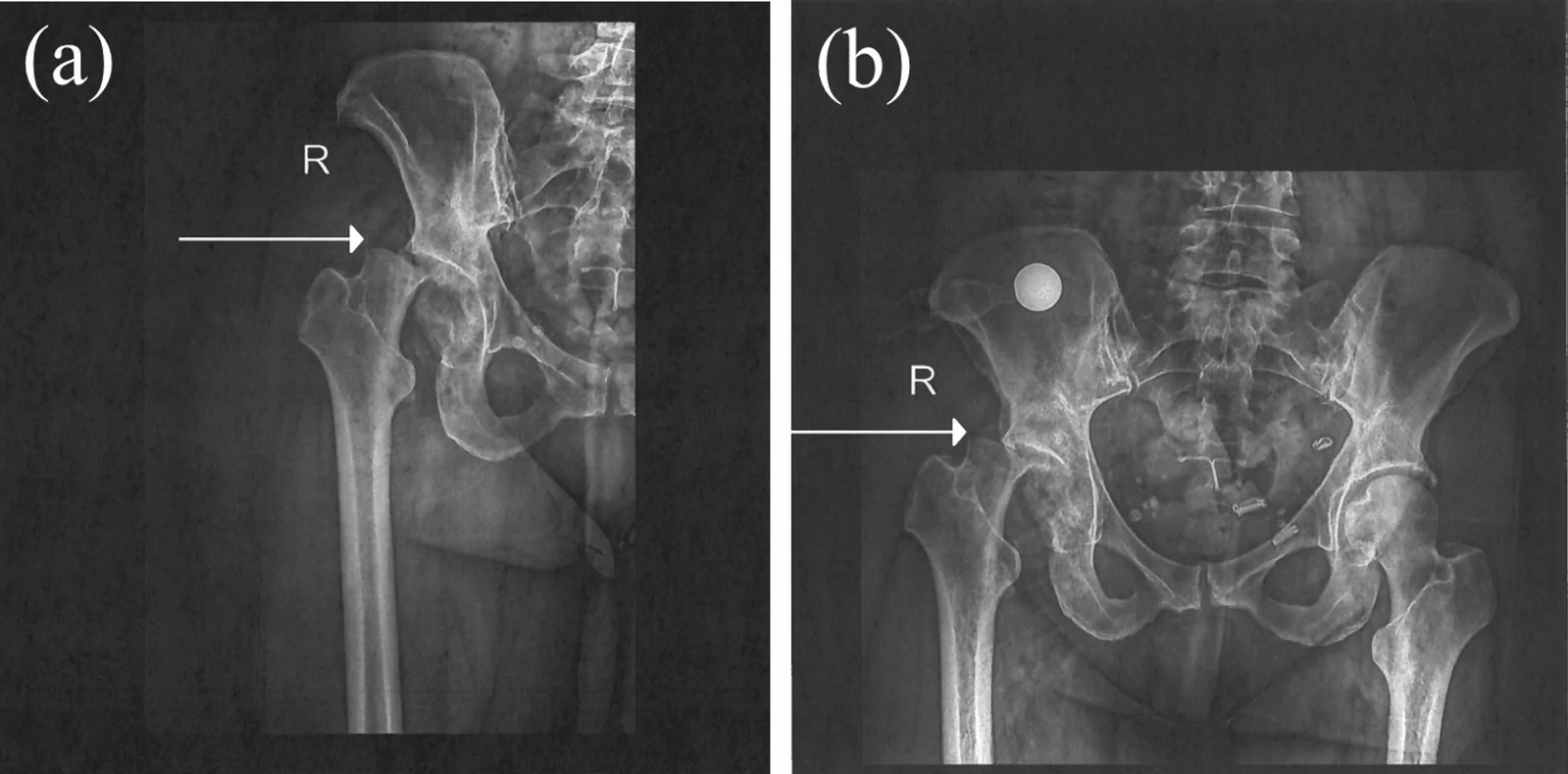
Right hip radiographs approximately six weeks following intra-articular corticosteroid injection (a) Anteroposterior radiograph of the right hip and (b) anteroposterior radiograph of the pelvis demonstrating marked interval progression with severe destruction and near-complete collapse of the right femoral head (arrows) compared with prior radiographs.

The patient’s rapid radiographic progression, severe pain, and increasing functional impairment prompted consideration for an urgent total hip arthroplasty despite the recent corticosteroid injection. The patient subsequently decided to undergo right total hip arthroplasty. Intraoperative findings demonstrated substantial degradation of the femoral head with only a residual stump of the femoral neck and advanced chondral degeneration. Histopathologic evaluation of the femoral head was not available. At postoperative follow-up, the patient reported substantial improvement in pain and ambulatory function. Follow-up radiographs shown in Figure 4 demonstrate the right total hip arthroplasty without evidence of acute complication. The patient’s clinical course, imaging progression, and treatment timeline are summarized in Table 1.

**Figure 4:**
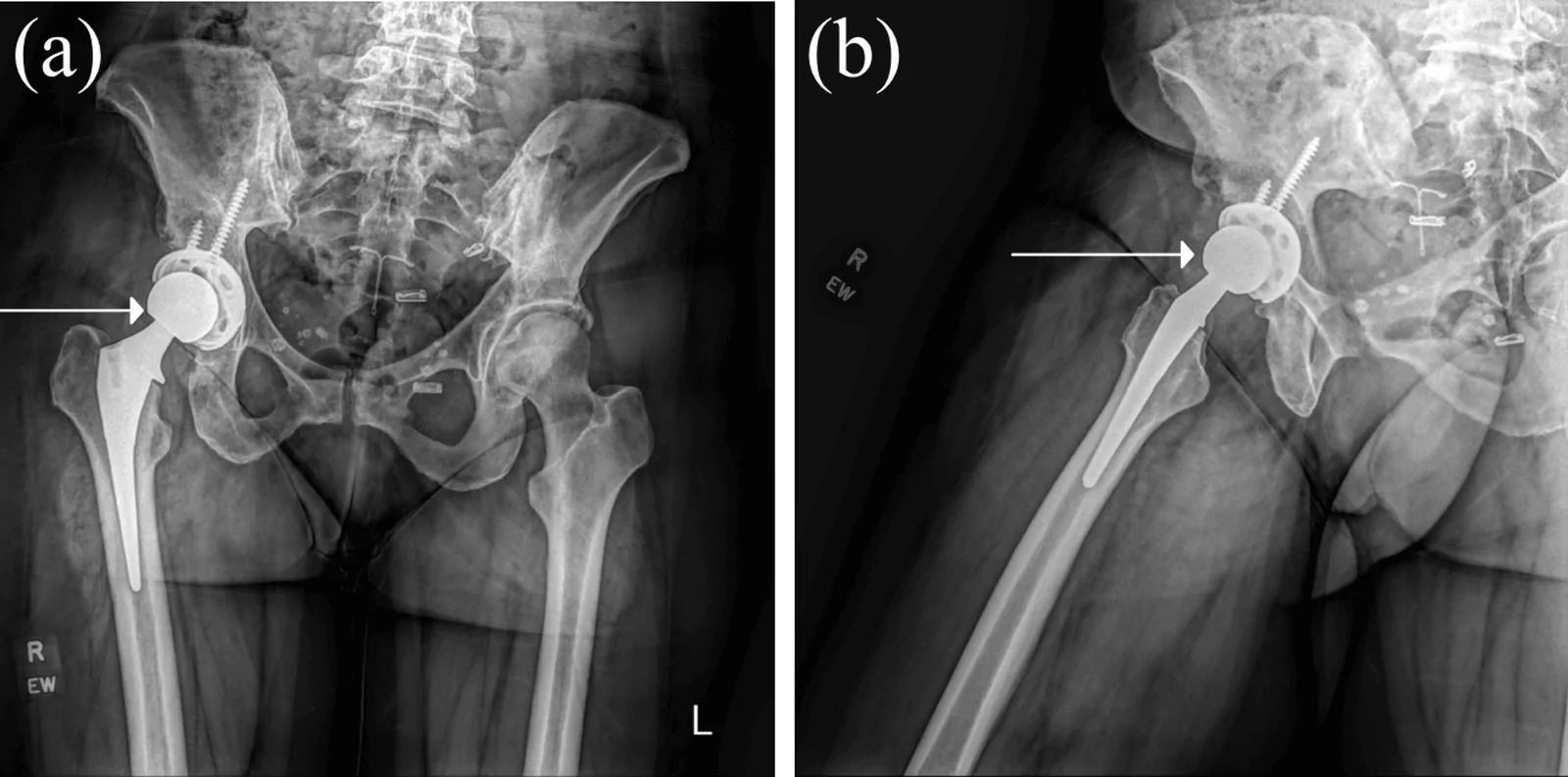
Right hip radiographs approximately six weeks following total hip arthroplasty (a) Anteroposterior radiograph of the pelvis and (b) frog-leg lateral radiograph of the right hip demonstrating right total hip arthroplasty components in satisfactory position (arrows), without evidence of postoperative complication.

**Table 1:** Timeline of clinical presentation, imaging progression, intervention, and postoperative outcome FABER: Flexion, abduction, and external rotation; FADIR: Flexion, adduction, and internal rotation.

Timepoint	Clinical and imaging findings
Initial presentation	Two-month history of progressive right groin pain radiating anteriorly to the knee. Positive log-roll test with mildly decreased right hip range of motion.
Pre-injection imaging	Radiographs demonstrated superior femoral head flattening, severe right hip osteoarthritis, and lateralization of the femoral head without evidence of frank collapse or subchondral lucency (Figure 1). MRI was not obtained; therefore, occult osteonecrosis or subchondral insufficiency fracture could not be excluded.
Intra-articular injection	Following failure of conservative treatments, the patient underwent fluoroscopy-guided intra-articular hip injection consisting of 80 mg of triamcinolone, 1.5 mL of 0.5% ropivacaine, and 1.5 mL of 2% lidocaine.
10 days post-injection	Worsening right groin pain and weakness with positive FABER, FADIR, and C-sign. Radiographs demonstrated interval deformity of the right femoral head compared with the pre-injection imaging (Figure 2).
~6 weeks post-injection	Markedly worsening pain and difficulty with ambulation. Radiographs demonstrated rapidly progressive, near-complete femoral head destruction (Figure 3). CT confirmed extensive destruction with joint effusion, capsular calcifications, and extension into the iliopsoas bursa.
Surgical intervention	Due to rapid structural deterioration and functional decline, the patient underwent urgent right total hip arthroplasty. Intraoperative findings included substantial femoral head degradation and advanced chondral degeneration. Histopathologic evaluation was unavailable.
~6 weeks postoperative	Substantial improvement in pain and ambulation. Follow-up radiographs demonstrated right total hip arthroplasty without evidence of complication (Figure 4).

## Discussion

This case demonstrates rapidly progressive femoral head destruction following a single intra-articular corticosteroid injection in a patient with pre-existing advanced radiographic hip osteoarthritis. The observed process is best characterized as rapidly progressive femoral head destruction based on the clinical course and serial radiographic findings rather than confirmed osteonecrosis because neither pre-injection MRI nor histopathologic confirmation was available. Although corticosteroid-associated osteonecrosis is more commonly associated with prolonged systemic exposure or higher cumulative doses, rapid femoral head collapse and osteonecrosis following a single intra-articular corticosteroid injection have been reported [6,7,10]. In this case, initial radiographs demonstrated advanced osteoarthritis and pre-existing superior femoral head flattening without radiographic evidence of frank collapse or subchondral lucency. Within 10 days after receiving an intra-articular injection containing 80 mg of triamcinolone, interval femoral head deformity was apparent, followed by near-complete destruction of the femoral head approximately six weeks after the injection. This unusually rapid radiographic progression is the principal distinguishing feature of the case. 

Previously reported cases have generally described longer intervals between injection and structural deterioration. Yamamoto et al. reported rapid femoral head collapse within three months of injection, with subsequent histopathologic evidence of osteonecrosis [6]. Al-Omari et al. described femoral head osteonecrosis following a single intra-articular corticosteroid injection and emphasized the rarity of this complication [10]. They noted that at the time of their study, only five cases of avascular necrosis from a single intra-articular steroid injection were reported. Similarly, Ahmed et al. reported two patients who developed destructive osteonecrosis 11 and 14 weeks after a single intra-articular corticosteroid injection and ultimately required total hip arthroplasty [7]. More broadly, rapidly progressive osteoarthritis has been reported following intra-articular hip corticosteroid injections [9]. A systematic review and meta-analysis estimated the incidence of rapidly progressive osteoarthritis following hip corticosteroid injection at approximately 6%. However, substantial variation in definitions and study methodology limits interpretation of this estimate [9]. 

Several possible processes could account for the rapid structural deterioration observed in this patient. Rapidly progressive osteoarthritis represents an important alternative or overlapping explanation for the findings in this patient, particularly given her severe pre-existing osteoarthritis. Occult pre-existing osteonecrosis and subchondral insufficiency fracture also cannot be excluded because MRI was not obtained before the injection, and both conditions may be radiographically occult early in their course. Infection represents another potential cause of rapidly destructive hip disease; however, objective data regarding an infectious evaluation were not available. Consequently, the relationship between intra-articular corticosteroid injection and subsequent joint destruction also remains uncertain. A prior matched-pair cohort study controlling for osteoarthritis severity and excluding patients with pre-existing osteonecrosis found no significant difference in short-term osteoarthritis progression or new femoral head collapse between patients who received corticosteroid injections and matched controls [11]. Similarly, a large propensity-matched cohort comparing corticosteroid with hyaluronic acid hip injections found no significant difference in the subsequent incidence of osteonecrosis [12]. These results suggest that some cases of osteonecrosis that are diagnosed following corticosteroid injection may reflect the natural progression of underlying hip disease rather than a direct adverse effect of corticosteroids [12]. Therefore, the temporal association observed in this case is insufficient to establish a causal relationship between the injection and subsequent femoral head destruction.

Despite these limitations, the marked radiographic deterioration over approximately six weeks following a single intra-articular corticosteroid injection warrants clinical attention. Rapidly worsening pain, inability to bear weight, or substantial functional decline following an injection should prompt repeat clinical and radiographic evaluation. In patients with symptoms disproportionate to radiographic findings or concern for occult osteonecrosis or subchondral insufficiency fracture, MRI should be considered in addition to serial radiographs. Early recognition of rapid structural deterioration may facilitate timely orthopedic evaluation and appropriate management.

## Conclusions

This case describes rapidly progressive femoral head destruction temporally associated with a single intra-articular corticosteroid injection. The patient progressed from advanced pre-existing osteoarthritis with superior femoral head flattening, but without definitive subchondral collapse or destructive loss of femoral head architecture, to near-complete femoral head destruction within approximately six weeks, ultimately requiring total hip arthroplasty. The temporal association does not establish causality, and alternative or pre-existing processes remain possible.

Clinicians should remain alert to rapidly worsening pain, declining function, or inability to bear weight following intra-articular hip corticosteroid injections. When symptoms are disproportionate to radiographic findings or concern exists for occult osteonecrosis or subchondral insufficiency fracture, MRI should be considered. Prompt reassessment of unexpected clinical deterioration may facilitate earlier recognition and management of rapidly progressive hip destruction.
